# Leg length discrepancy after in situ fixation with screw for slipped capital femoral epiphysis

**DOI:** 10.1038/s41598-022-06347-9

**Published:** 2022-02-10

**Authors:** Sungmin Kim, Kun-Bo Park, Hyun Woo Kim, Jong Eun Kim, Hoon Park

**Affiliations:** 1 Department of Orthopedic Surgery, Chonnam National University Medical School and Hospital, 42 Jebong-ro, Dong-gu, Gwangju, 61469 Republic of Korea; 2 Division of Orthopaedic Surgery, Severance Children’s Hospital, Yonsei University College of Medicine, Seoul, 03722 Korea; 3 Department of Orthopaedic Surgery, Gangnam Severance Hospital, Yonsei University College of Medicine, Seoul, 06273 Korea

**Keywords:** Diseases, Health care, Medical research, Risk factors

## Abstract

Although leg length discrepancy (LLD) commonly occurs following in situ fixation with screws for slipped capital femoral epiphysis (SCFE), the literature regarding this issue is scarce. The purpose of this study was to evaluate the degree of LLD in patients who had been treated with in situ fixation with screws and to identify the risk factors for the development of LLD. We retrospectively reviewed 44 patients (mild slip 24, moderate slip, 20) who were treated with in situ fixation with screws for SCFE. The mean age at surgery was 12.2 years and the mean follow-up period was 6.9 years. We investigated the relationship between the final LLD, articulotrochanteric distance difference (ATDD) at skeletal maturity, and various clinical and radiographic parameters using linear regression analysis. The mean values of LLD and ATDD were 13.1 and 11.1 mm, respectively. The LLD and ATDD was significantly higher in patients with moderate slips than in those with mild slips. The degree of slip angle was associated with the degree of LLD only. While there was no significant factor affecting the LLD in moderate slips, younger age and a larger degree of slip angle were associated with the degree of LLD. The degree of slip was the only factor that affected LLD in patients with mild or moderate SCFE who underwent threaded screw fixation. Age at surgery was not associated with LLD, and there were no factors related to the degree of LLD in mild slip. Monitoring for LLD may only be necessary for patients with moderate slip who are treated with in situ screw fixation.

## Introduction

Slipped capital femoral epiphysis (SCFE) is a well-known hip disorder occurring in adolescence, whereby the epiphysis is displaced posteroinferiorly to the metaphysis through the physis^1^. The overall incidence ranges from 1 to 10 per 100,000 children, with an onset at age 11–13 years in girls and 13–15 years in boys^2^. The goal of treatment is to prevent additional slippage, and the most widely used treatment for SCFE is in situ fixation without attempts at reduction of the deformity^3^. Possible complications include osteonecrosis, chondrolysis, impingement syndrome, leg length discrepancy (LLD), and degenerative joint disease.

LLD may manifest as shortening of the affected limb from the deformity of the femoral head due to slip of the epiphysis. This is exacerbated further by shortening of the affected limb from proximal femoral growth disturbance by in situ fixation with screws. Thus, various degrees of LLD commonly occur after screw fixation for SCFE. As SCFE occurs before skeletal maturity, LLD can increase with age. However, to the best of our knowledge, only one study has reported the magnitude of LLD associated with SCFE following in situ fixation and the factors affecting the development of LLD^4^. In that study, the severity of slip was related to the magnitude of LLD. Interestingly, despite SCFE occurring in skeletally immature patients, the study reported that the age at surgery was not correlated with the magnitude of LLD.

Therefore, the primary aim of this study was to evaluate the degree of LLD in patients who had been treated with in situ fixation with screws for SCFE and to identify the risk factors for the development of LLD. We hypothesized that age at surgery would affect the degree of LLD in patients who underwent in situ fixation with screws for SCFE.

## Results

The mean slip angle was 30.1 (9.5–59.8) degrees. Twenty-four hips were classified as having mild slips, and 20 hips showed moderate slips. None of the patients had severe slips. The mean values of LLD and ATDD at the latest follow-up were 13.1 mm (5–30 mm) and 11.1 mm (2–27 mm), respectively. Fifteen patients had a small LLD, 14 had a moderate LLD, and 15 had a large LLD.

**Figure 1:**
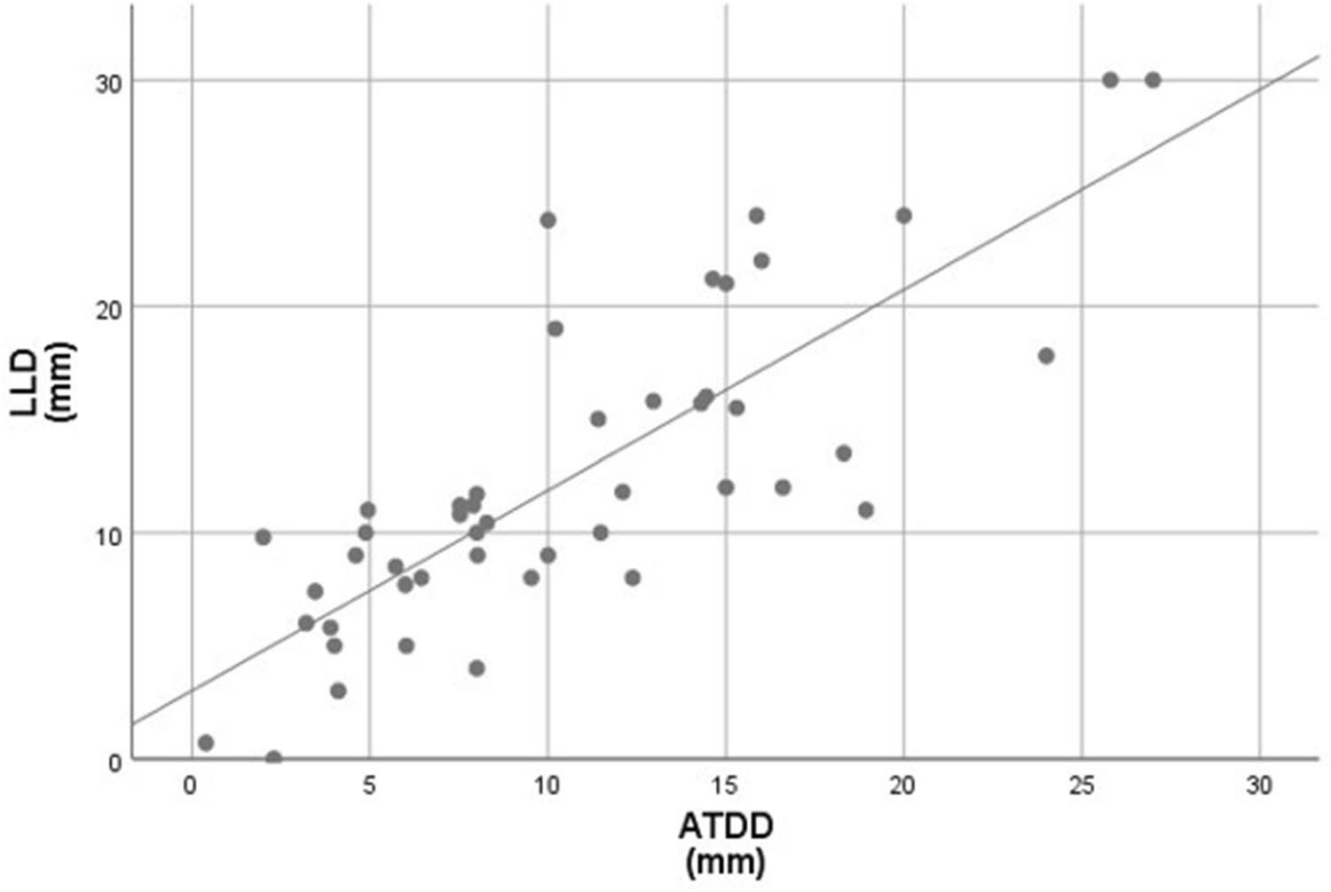
Relationship between leg length discrepancy (LLD) and articulotrochanteric distance difference (ATDD) was analyzed with Pearson correlation coefficients. LLD was highly correlated with ATDD (r = 0.776, p < 0.001).

Comparisons of LLD and ATDD between subgroups divided by clinical or radiographic variables are shown in Table 1. There were significant differences in both LLD and ATDD between patients with mild and moderate slips (p < 0.001 and p < 0.001, respectively). There was no difference in both LLD and ATDD between the groups divided by each variable, except for the slip severity. LLD was highly correlated with ATDD (Fig. 1, r = 0.776, p < 0.001).

**Table 1:** Comparison of leg length discrepancy and articulotrochanteric distance difference according to clinical and radiological variables.

	Patients	Leg length discrepancy	*p*-value	Articulotrochanteric distance difference	*p*-value
**Age at surgery, year**			0.599		0.494
< 11	10	12.1 (7.4–21.2)		10.3 (2.0–18.3)	
11–13	20	14.3 (5.0–30.0)		12.3 (3.9–25.8)	
> 13	14	12.3 (5.0–30.0)		9.9 (3.2–27.0)	
**Sex**			0.902		0.723
Male	32	13.2 (5.0–30.0)		11.3 (3.2–27.0)	
Female	12	12.9 (5.0–24.0)		10.6 (2.0–24.0)	
**Symptom duration**			0.512		0.113
Acute	27	12.6 (5.0–24.0)		10.0 (2.0–20.0)	
Chronic	17	13.9 (7.4–30.0)		12.9 (3.5–27.0)	
**Stability of slip**			0.154		0.902
Stable	39	12.6 (5.0–30.0)		11.2 (2.0–27.0)	
Unstable	5	17.1 (5.8–23.8)		10.8 (3.9–15.3)	
**Severity of slip**			< 0.001		< 0.001
Mild	24	9.7 (5.0 – 15.8)		8.3 (2.0–18.3)	
Moderate	20	17.3 (5.0–30.0)		14.5 (4.0–27.0)	

Values are expressed as the means (range) unless otherwise indicated.

In the univariable linear regression analysis of all included patients, LLD was associated with the degree of slip angle (0.279, 95% CI = 0.177 to 0.381, p < 0.001), indicating that as the slip severity increased, LLD increased. ATDD was also related to the degree of slip angle (0.233, 95% CI = 0.129 to 0.336, p < 0.001). Other factors showed no significant association with either LLD or ATDD (Table 2).

**Table 2:** Univariable linear regression analysis of LLD and ATDD in all cohorts.

Factor	LLD		ATDD	
	Coefficient*	*p*-value	Coefficient*	*p*-value
Age at surgery	− 0.043 (− 1.354 to 1.268)	0.948	− 0.185 (− 1.421 to 1.052)	0.765
**Sex**				
Male vs. female	0.275 (− 4.203 to 4.753)	0.902	0.746 (− 3.474 to 4.967)	0.723
BMI	− 0.099 (− 0.553 to 0.355)	0.663	0.029 (− 0.400 to 0.458)	0.893
**Symptom duration**				
Acute vs. chronic	1.337 (− 2.739 to 5.412)	0.512	3.006 (− 0.745 to 6.758)	0.113
**Stability of slip**				
Stable vs. unstable	4.410 (− 1.723 to 10.543)	0.154	− 0.364 (− 6.294 to 5.566)	0.902
Slip angle	0.279 (0.177 to 0.381)	< 0.001	0.233 (0.129 to 0.336)	< 0.001

*CI* confidence interval, *LLD* leg length discrepancy, *ATDD* articulotrochanteric distance difference.

*Values are given as coefficients with the 95% CI in parentheses.

**Figure 2:**
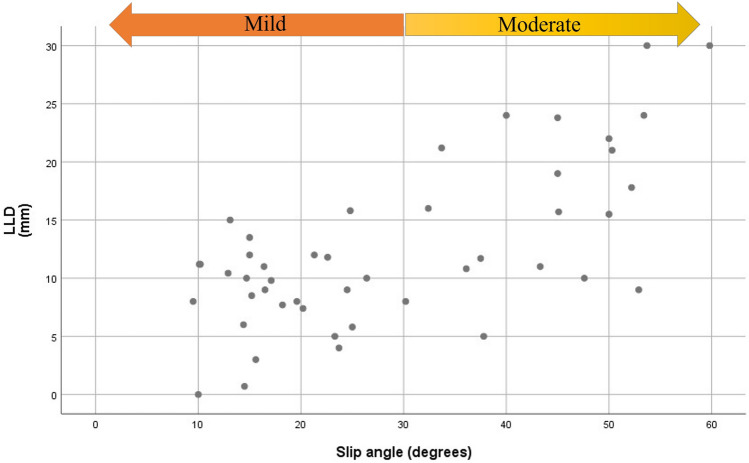
Scatterplot shows the relationship between slip angle and leg length discrepancy (LLD). The final degree of LLD was less than 1.5 cm in almost all patients with mild slip.

In the subgroup analysis of patients with mild slip, there were no statistically significant variables affecting LLD (Table 3). However, in the subgroup analysis of patients with moderate slip (Table 4), multivariate analysis showed that younger age (-2.995, 95% CI = − 5.048 to − 0.941, p = 0.007) and a larger slip angle (0.561, 95% CI = 0.244 to 0.878, p = 0.002) were significantly associated with larger LLD. The relationship between slip angle and LLD is shown in Fig. 2. The scatter plot indicates that the degree of LLD was less than 1.5 cm in almost all patients with mild slips.

**Table 3:** Linear regression analysis of leg length discrepancy in patients with mild slip.

Factor	Univariable	
	Coefficient^a^	*p*-value
Age at surgery	− 0.486 (− 1.259 to 0.287)	0.206
**Sex**		
Male vs. female	3.077 (0.007 to 6.146)	0.060
BMI	− 0.099 (− 0.451 to 0.254)	0.567
**Symptom duration**		
Acute vs. chronic	0.871 (− 1.993 to 3.734)	0.535
**Stability of slip**		
Stable vs. unstable	− 4.084 (− 10.080 to 1.921)	0.172
Slip angle	− 0.120 (− 0.363 to 0.123)	0.317

*CI* confidence interval.

^a^The values are given as coefficients, with the 95% CI in parentheses.

**Table 4:** Linear regression analysis of leg length discrepancy in patients with moderate slip.

Factor	Univariable		Multivariable	
	Coefficient^a^	*p*-value	Coefficient^a^	*p*-value
Age at surgery	− 2.152 (− 4.761 to 0.457)	0.100	− 2.995 (− 5.048 to − 0.941)	0.007
**Sex**				
Male vs. female	2.374 (− 4.638 to 9.385)	0.486		
BMI	− 0.120 (− 0.781 to 0.541)	0.707		
**Symptom duration**				
Acute vs. chronic	− 2.974 (− 9.818 to 3.870)	0.373		
**Stability of slip**				
Stable vs. unstable	3.249 (− 5.308 to 11.807)	0.435		
Slip angle	0.454 (0.082 to 0.826)	0.019	0.561 (0.244 to 0.878)	0.002

*CI* confidence interval.

^a^The values are given as coefficients with the 95% CI in parentheses.

## Discussion

Traditionally, SCFE has been treated by in situ fixation of the femoral head with a single cannulated screw to inhibit further slippage^5,6^. In situ fixation with screws can consequentially cause a physeal arrest, which leads to a relative shortening of the femoral neck^7–9^ and subsequent LLD^4,10^. As SCFE usually occurs before skeletal maturity, we can assume that the degree of LLD may be greater in younger patients. In our study, we found that slip angle severity was the only factor associated with the degree of LLD. Contrary to our hypothesis, age at the time of surgery was not associated with the final LLD.

Interestingly, there was no relationship between age at surgery and LLD. We found that younger age was associated with a larger LLD, but only in patients with moderate slips. Our findings were different from those of a previous study^4^. In that study, although there was no relation between the age at surgery and the degree of LLD, the patients who underwent in situ pinning at the older age often had an increase in LLD compared with the patients who underwent surgery at a younger age. They assumed that the reason might be the increased slip angle according to age^4^. A possible explanation for this discrepancy is that the degree of growth remaining in the proximal femur in patients with SCFE is not as high as we assume, although the average age at onset of SCFE is 12 years^11,12^, which is a period of rapid growth and considerable growth potential remains. Although approximately 30% of the overall longitudinal growth of the femur occurs at the proximal end, by skeletal age 14 years in girls and 16 years in boys, virtually all the growth occurs from the distal physis^13^. Some studies have also shown that closure of the proximal femoral physis in most cases of SCFE would not be expected to cause significant retardation of growth^14,15^. We postulated that the contribution of the proximal femoral physis to total leg length is not much in this age group and the growth arrest by screw fixation may not have a significant impact on the growth of the proximal femur.

As anticipated, the severity of the slip angle was associated with LLD in all cohorts. We were unable to find a significant relationship between LLD and other factors. These results were consistent with those of a previous study^4^, which showed that the severity of the slip angle itself has the greatest effect on the degree of LLD. Interestingly, the slip severity was only significantly associated with LLD in patients with moderate slips, but not in patients with mild slips. This result could be attributed to remodeling of the femoral head. It has been demonstrated that remodeling of femoral head occurs following screw fixation around physis^16–22^, even after threaded screw fixation^9,23^. Some authors described that remodeling of proximal femur occurred in patients with mild SCFE were treated by fixation in situ using a modified screw^16,19^. Örtegren et al.^16^ insisted that remodeling potential may be greater in patients with mild slip than in patients with moderate or severe slip. Other studies have shown that a slippage angle of approximately 30 to 35 degrees is the threshold at which FAI appears more regularly^24,25^, suggesting remodeling of the femoral head in patients with mild slips.

There were no statistically significant variables affecting LLD in mild slips, and the degree of LLD at skeletal maturity was less than 1.5 cm in almost all patients with mild slips. LLD less than 20 mm is often asymptomatic and represents a normal variant. Surgical correction is usually recommended for LLD greater than 15–20 mm^26^. Thus, our results indicate that monitoring for LLD in mild slip may be unnecessary. Moreover, the alternative growth-preserving implant for in situ pinning^5,18^, which has been a longstanding interest in treating SCFE, may be unnecessary for mild slips. It seems reasonable to carefully monitor the LLD in patients with moderate slips.

Our results showed a significant relationship between LLD and ATDD. ATDD has been used to study trochanteric overgrowth with abduction insufficiency after congenital dislocation of the hip, Legg-Calve-Perthes disease, infantile coxa vara, and patients with SCFE after screw fixation^27–29^. Ordeberg et al.^29^ suggested that reduction in ATD could be a useful finding for showing damage to the subcapital growth plate in patients with SCFE. Kim et al.^4^ found a significant relationship between LLD and ATDD in their regression analysis. We agree that these findings support the use of AP pelvic radiography as the initial imaging study to assess LLD by measuring the difference in ATDD between the two sides in patients with SCFE. These findings support the use of ATDD as a useful marker for studying growth disturbances of the proximal femur in patients with SCFE^4,29^.

Our study had several limitations. First, a relatively small number of patients were included in the study. For the accurate evaluation of LLD at skeletal maturity, 15 patients with remaining growth and 22 patients with bilateral involvement were excluded. We believe that these exclusions would have reduced biases. Second, 5 patients with severe slips were excluded from our study due to osteonecrosis. There is a possibility that the inclusion of severe slips may affect the results of analysis. However, severe slip is rare and other surgical treatments such as Dunn procedure have recently been applied, making it unlikely to be included in this study. Lastly, there are also limitations and potential implications associated with the retrospective study design, including the variability of follow-up periods.

In conclusion, the slip degree was related to LLD in patients with mild or moderate SCFE who underwent threaded screw fixation. Contrary to conventional belief, age at surgery was not associated with LLD, and there was no factor related to the degree of LLD in mild slips. Monitoring for LLD may be necessary only for patients with moderate slip who are treated with in situ screw fixation.

## Methods

Our Institutional Review Board of the Gangnam Severance Hospital (IRB No. 3-2020-0306) approved this retrospective study and waived the need of receiving informed consent from the patients. All methods were performed in accordance with the relevant guidelines and regulations. We identified all consecutive patients with a diagnosis of SCFE who had undergone in situ fixation with screws at our department. The inclusion criteria were as follows: (1) in situ fixation with screws, (2) unilateral SCFE, (3) presence of LLD > 5 mm^30^, and (4) radiologic follow-up until growth maturity. Patients were excluded for the following reasons: a history of other surgical treatments such as osteotomy or epiphysiodesis, complications such as avascular necrosis or chondrolysis, or inadequate follow-up radiographs available for review.

We initially identified 89 pediatric patients who were treated for unilateral SCFE. From the identified sample population, 10 patients underwent prophylactic fixation on the contralateral side of the hip, and 13 patients developed a subsequent contralateral slip. Additionally, 19 patients were excluded because of postoperative complications (avascular necrosis, n = 3), subsequent operation (epiphysiodesis, n = 2), or no follow-up until growth maturity at the most recent follow-up (n = 14). Among the remaining 47 patients, 3 patients were excluded because they showed LLD of < 5 mm on the latest radiograph.

A total of 44 patients were included in the study. The mean age at surgery was 12.2 (8–15.2) years. The average age at the final follow-up was 19.2 years (18 to 24.2 years). There were 32 boys and 12 girls. The mean body mass index (BMI) was 26.7 (19.6 38.6). The mean follow-up period was 6.9 years (3.1 to 11.4 years).

All surgeries were performed using the same surgical technique. The patient was positioned supine on a fracture table with the contralateral lower limb in the hemilithotomy position. A 2–3 cm longitudinal incision was made over the anterolateral aspect of the proximal part of the femur. Under fluoroscopic guidance, a guidewire was advanced freehand into the “center-center” of the epiphysis, stopping approximately 3 mm short of the articular surface. The guidewire was overdrilled, and a 6.5- or 7.0-mm partially threaded cannulated screw of appropriate length was inserted into the epiphysis. The femur was brough through a full range of internal–external rotation under fluoroscopy to confirm that the screw did not penetrate the joint. The patients were mobilized with partial weight-bearing on the affected side for 4 weeks postoperatively. They were followed up with serial radiographs at approximately 6 months to 1-year intervals to monitor the contralateral hip until skeletal maturity. None of the patients had postoperative infections or hardware failure.

**Figure 3:**
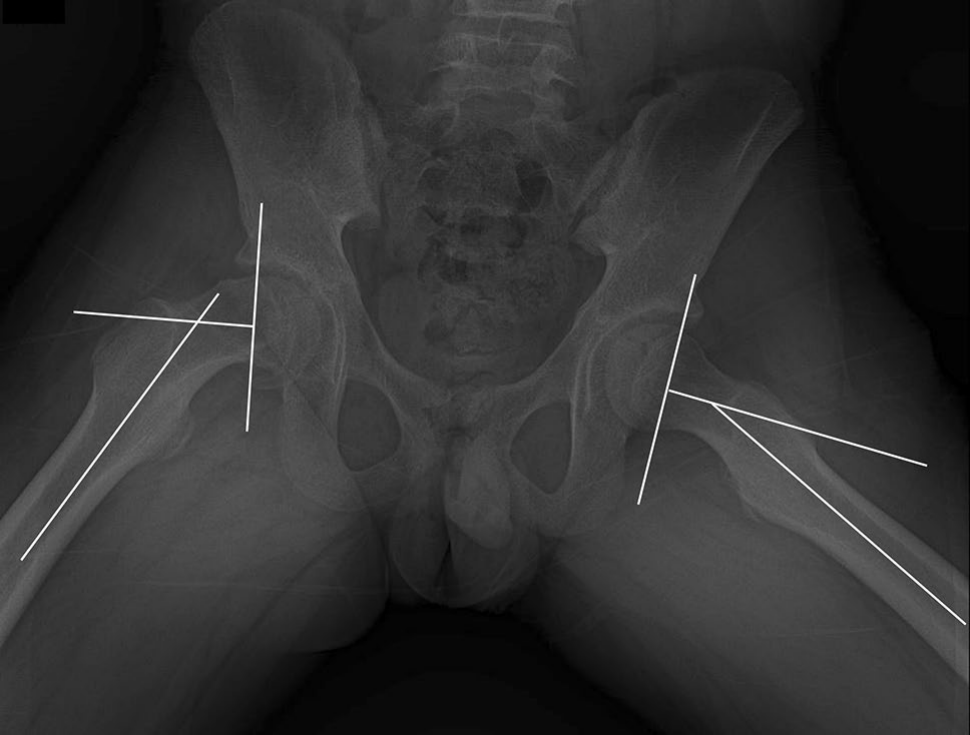
Measurement of the Southwick angle on a frog-leg lateral radiograph.

The following data were obtained: age at surgery, sex, BMI, symptom duration, stability and severity of the slip, and duration of follow-up. Patients were divided based on the symptom duration before diagnosis. Chronic slips are those causing symptoms for a period of at least 3 weeks, whereas acute slips are those that are symptomatic for < 3 weeks^12^. Patients were classified as either stable or unstable based on their ability to bear weight, even with crutches^1^. The severity of the slip was determined using the Southwick (SW) angle, measured as the angle between the proximal femoral physis and the femoral shaft on a frog-leg lateral radiograph (Fig. 3)^31^. The difference between these two angles obtained at the affected and unaffected sides is referred to as SW angles. A difference of < 30 degrees was graded as mild, a difference of 30 to 50 degrees moderate, and more than 50 degrees were graded as severe^32^.

**Figure 4:**
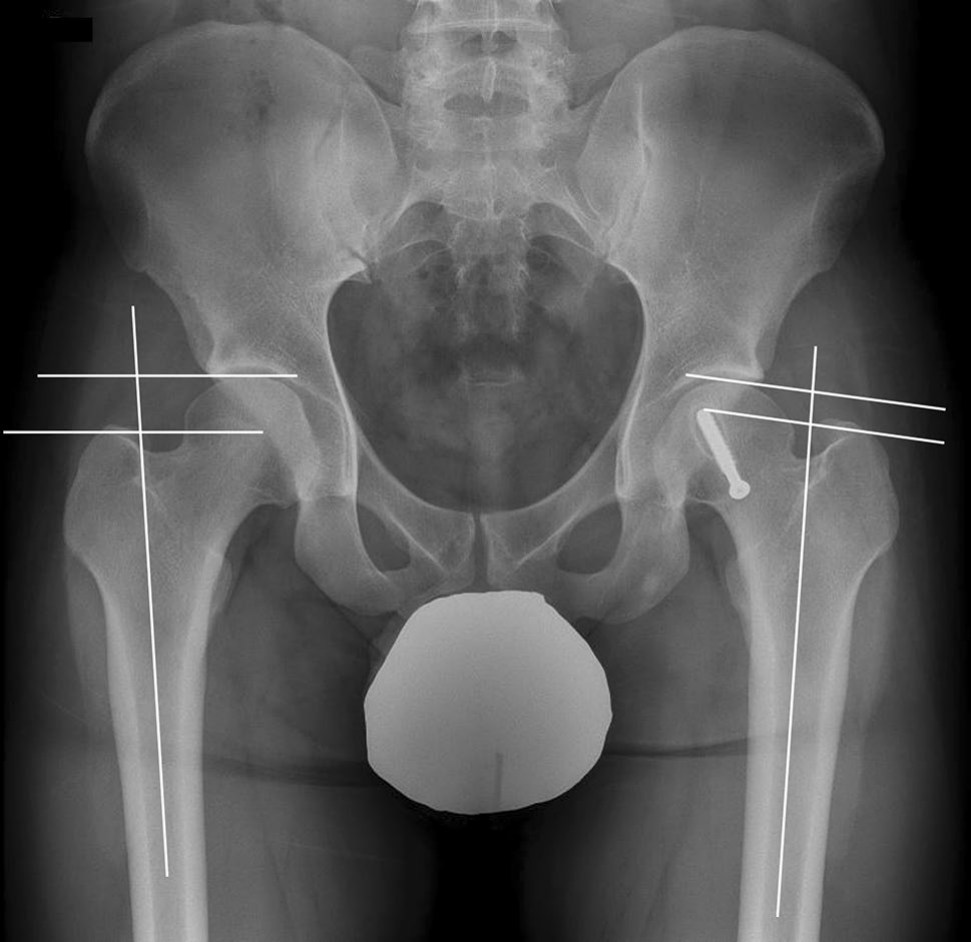
Measurement of the articulotrochanteric distance on supine anteroposterior radiograph of the hip. Articulotrochanteric distance difference was calculated as healthy side minus the involved side.

LLD was determined on full-length standing anteroposterior hip-to-ankle radiographs at skeletal maturity. The leg length was measured from the center of the femoral head to the superior border of the talus. LLD was recorded as a positive value when the affected side was longer than the unaffected side. LLDs were stratified into four groups based on cutoffs from previous literature^30^: no LLD (< 5 mm), small LLD (5 mm ≤ LLD < 10 mm), moderate LLD (10 mm ≤ LLD < 15 mm), and large LLD (≥ 15 mm). The articulotrochanteric distance (ATD) was also measured from supine anteroposterior hip projections at skeletal maturity (Fig. 4)^29^. Perpendiculars to the longitudinal axis of the femoral diaphysis was drawn at the level of the proximal limit of the femoral head and at the tip of the greater trochanter. The ATD was given a positive sign if the proximal limit of the femoral head was situated proximally to the tip of the greater trochanter; otherwise, it was negative. Articulotrochanteric distance difference (ATDD) was calculated as the healthy side minus the side with SCFE. Radiologic assessments were performed by two orthopedic residents who were blinded to the study details.

### Statistical analysis

To compare the LLD and ATDD between the two groups regarding clinical characteristics and radiologic measurements, the 2-sample t-test was used for continuous variables, and the x^2^ test was employed to compare categorical variables. To compare the LLD and ATDD between the three subgroups regarding age at surgery, an analysis of variance for continuous variables was used. The relationship between LLD and ATDD was analyzed using the Pearson correlation coefficients. To examine the effects of several variables on LLD and ATDD, we used a univariable linear regression analysis. In each univariate model, all potential risk factors were analyzed, and variables identified as significant in the univariate analysis, with a p-value < 0.1, as well as clinical variables were included in the multivariate analysis. Statistical analyses were performed using SPSS® version 25.0 (SPSS, Chicago, IL, USA), with significance defined as p < 0.05.

### Ethical approval

This study was approved by the institutional review board of the Gangnam Severance Hospital, Seoul, Korea (IRB number; 3-2020-0306).

### Consent to participate

The IRB of our hospital waived the need of receiving informed consent from the patients; our research involved no more than a minimal risk to our subjects, and we used the existing medical records and the radiographs.

## Data Availability

All data generated or analyzed during this study are available from the corresponding author upon reasonable request.
